# Optimal allocation of physicians improves accessibility and workload disparities in stroke care

**DOI:** 10.1186/s12939-023-02036-9

**Published:** 2023-11-07

**Authors:** Kazuki Ohashi, Toshiya Osanai, Kyohei Bando, Kensuke Fujiwara, Takumi Tanikawa, Yuji Tani, Soichiro Takamiya, Hirotaka Sato, Yasuhiro Morii, Tomoki Ishikawa, Katsuhiko Ogasawara

**Affiliations:** 1 Faculty of Health Sciences, Hokkaido University, N12-W5, Kita-ku, Sapporo, 060-0812 Japan; 2 Department of Neurosurgery, Faculty of Medicine, Graduate School of Medicine, Hokkaido University, N15-W7, Kita-ku, Sapporo, 060-8638 Japan; 3 Graduate School of Health Sciences, Hokkaido University, N12-W5, Kita-ku, Sapporo, 060-0812 Japan; 4 Graduate School of Commerce, Otaru University of Commerce, 3-5-21, 047-8501 Otaru, Midori Japan; 5 Faculty of Health Sciences, Hokkaido University of Science, 7-15-4-1, Maeda, Teine-ku, Sapporo, 006- 8585 Japan; 6 Department of Medical Informatics and Hospital Management, Asahikawa Medical University, E2-1-1-1, 078-8510 Asahikawa, Midorigaoka Japan; 7 Department of Neurosurgery, Otaru General Hospital, 1-1-1, Wakamatsu, Otaru, 047-8550 Japan; 8 Department of Neurosurgery, Asahikawa Medical University, E2-1-1-1, Midorigaoka, Asahikawa, 078- 8510 Japan; 9 Center for Outcomes Research and Economic Evaluation for Health, National Institute of Public Health, 2-3-6, Minami, Wako, Japan; 10 Institute for Health Economics and Policy, 1-21-19, Toranomon, Minato-ku, 105-0001 Japan

**Keywords:** Two-step floating catchment area method, Quadratic programming, Potential crowdedness index, Spatial accessibility, Mechanical thrombectomy, Primary Stroke center

## Abstract

**Background:**

Inequalities in access to stroke care and the workload of physicians have been a challenge in recent times. This may be resolved by allocating physicians suitable for the expected demand. Therefore, this study analyzes whether reallocation using an optimization model reduces disparities in spatial access to healthcare and excessive workload.

**Methods:**

This study targeted neuroendovascular specialists and primary stroke centers in Japan and employed an optimization model for reallocating neuroendovascular specialists to reduce the disparity in spatial accessibility to stroke treatment and workload for neuroendovascular specialists in Japan. A two-step floating catchment area method and an inverted two-step floating catchment area method were used to estimate the spatial accessibility and workload of neuroendovascular specialists as a potential crowdedness index. Quadratic programming has been proposed for the reallocation of neuroendovascular specialists.

**Results:**

The reallocation of neuroendovascular specialists reduced the disparity in spatial accessibility and the potential crowdedness index. The standard deviation (SD) of the demand-weighted spatial accessibility index improved from 125.625 to 97.625. Simultaneously, the weighted median spatial accessibility index increased from 2.811 to 3.929. Additionally, the SD of the potential crowdedness index for estimating workload disparity decreased from 10,040.36 to 5934.275 after optimization. The sensitivity analysis also showed a similar trend of reducing disparities.

**Conclusions:**

The reallocation of neuroendovascular specialists reduced regional disparities in spatial accessibility to healthcare, potential crowdedness index, and disparities between facilities. Our findings contribute to planning health policies to realize equity throughout the healthcare system.

**Supplementary Information:**

The online version contains supplementary material available at 10.1186/s12939-023-02036-9.

## Background

Inequality of access to healthcare is an ongoing challenge worldwide [1–4]. One of the causes of inequality of access to healthcare is physician maldistribution between urban and rural areas [5, 6]. Disparity in accessibility to healthcare and physician maldistribution or shortage is negatively correlated with public health outcomes [7, 8]. Moreover, the COVID-19 pandemic has emphasized that human resource shortage results in excessive workload and burnout among medical professionals [9, 10]. Therefore, the importance of balancing supply and demand in healthcare is more significant than ever before.

Methods for evaluating spatial accessibility (SA) to healthcare include provider-to-population ratio, distance or time to the nearest hospital, average distance or time to reachable hospitals, and the gravity model [11]. Luo and Wang expanded the gravity model and developed the two-step floating catchment area method (2SFCAM), which estimates SA to healthcare proportions of supply and demand [12]. The 2SFCAM, with the strength of estimating potential SA in healthcare, is commonly used in healthcare studies [4, 13]. A study by Wang et al. revealed that the inverted 2SFCAM could estimate the potential crowdedness index (PCI) [14]. It elucidated that PCI at their facility strongly correlated with actual discharged patients [15]. Thus, equalization of PCI as a workload at each facility or staff can equalize the workload of medical professionals with limited human resources. Some studies have introduced 2SFCAM and quadratic programming (QP), focusing on allocating hospital beds and primary care doctors to equalize accessibility to healthcare [16, 17]. However, to the best of our knowledge, no studies have focused on equalizing PCI to distribute the workload for medical professionals.

This study focused on PCI for mechanical thrombectomy (MT), the primary treatment for acute ischemic stroke. Several randomized controlled trials have indicated improved outcomes with the addition of thrombectomy instead of thrombolytic therapy alone [18], and future indications are expected to be expanded. The expansion of indications, that is, an increase in the number of implementations, directly leads to an increase in the workload of physicians. An immediate consequence of heightened workloads is hospital congestion, which may lead to delays in patient care, including MT. Prolonged periods of such conditions can deteriorate the work environment of physicians, potentially resulting in burnout and understaffing. This understaffing, when persistent, exacerbates regional disparities in access to MT – an issue already observed in Japan [19]. Equalizing the workload of physicians and improving access to MT are inevitable health policy challenges. Therefore, this study aimed to equalize the PCI and SA of MT using 2SFCAM and QP and describe the changes in PCI and the number of physicians at the prefecture level.

## Methods

### Study design

This was a cross-sectional simulation study using a public database.

### Study target

This study targeted neuroendovascular specialists (NES) at primary stroke centers and registered MT training hospitals in Japan. The number of NES and facilities in September 2021 was 1,605 and 662, respectively. Additionally, Japan’s 47 prefectures were used for geographic evaluation (Additional file 1).

### Data source and software

This study used an estimated population aged ≥ 65 years for 2020 based on the national census conducted in 2015, a mesh dataset (mesh size = 500 m × 500 m), and the address of medical facilities as a shape file [20], including PSC [21]. The certification criteria for PSC are described in Additional file 1. The NES and MT training hospitals (three hospitals) were identified from the website of The Japanese Society for Neuroendovascular Therapy [22]. The ArcGIS Geo Suite Network Road 2021 Japan version (Esri Japan, Sumitomo Denko, Japan) was used as the road network dataset. The distance matrix between the facilities and the central point of the mesh was created using ArcGISpro2.8 (ESRI Inc. Redlands, USA). R (4.1.1) and RStudio (2021.9.0.351) were used to conduct all statistical analyses, and QP was performed using the R package “quadprog” [23–25].

### SA index and PCI using 2SFCAM and inverted 2SFCAM

The 2SFCAM and inverted 2SFCAM calculated the SA index (Eqs. 1 and 2) and PCI (Eqs. 3 and 4), respectively. This study defined the population aged ≥ 65 years as the demand for MT, the number of NES at facilities as the supply for MT, and travel time within 120 min by car between the starting point and the facility as the reachable range. In addition, the exponential distance decay function was used to consider that facilities closer to the starting point were preferred (Eq. 5). The travel friction coefficient β in the distance decay function was set to 0.07, based on the transportation time (median: 10 min) for patients receiving MT in Japan [26]. In other words, when the travel time was 10 min, the weight was approximately 0.5. We conducted a sensitivity analysis to examine uncertainty by varying β from 0.07 to 0.02 in 0.01 increments (Additional file 1). This change in β reduces the impact of travel time on the results.

Equation 1:

$${R}_{j}=\frac{{S}_{j}}{{\sum }_{{d}_{ij}\in {d}_{0}}{D}_{i}*f\left({d}_{ij}\right)}$$

Equation 2:

$${A}_{i}={\sum }_{{d}_{ik}\in {d}_{0}}{R}_{k}*f\left({d}_{ik}\right)$$

Equation 3:

$${r}_{i}=\frac{{D}_{i}}{{\sum }_{{d}_{ij}\in {d}_{0}}{S}_{j}*f\left({d}_{ij}\right)}$$

Equation 4:

$${C}_{j}={\sum }_{{d}_{jl}\in {d}_{0}}{r}_{l}*f\left({d}_{jl}\right)$$

Equation 5:

$$f\left({d}_{ij}\right)=\left\{\begin{array}{c}{{e}^{-\beta *{d}_{ij}}, d}_{ij}<{d}_{0} \\ \\ 0, { d}_{ij}\ge {d}_{0}\end{array}\right.$$

where i and l are meshes; j and k are facilities; R is the supply per population aged ≥ 65 years; S is the number of NES at a facility; D is the population aged ≥ 65 years; A is the SA index; r is the demand per number of NES; C is PCI; β is the travel friction coefficient; d_ij_, d_ik_, and d_il_ are the travel times between the mesh and facility (min); *f* () is the distance decay function; and d0 is 120 min.

### Quadratic programming

The QP was performed according to the method described by Wang et al. [17]. The objective function was defined as the minimizing distribution of the demand-weighted SA index, which signified that the total NES was 1605, and the reallocation range of NES was 0 to 5 at a facility (Eq. 6).

Equation 6:

Objective function

$$minimize\sum _{i=1}{D}_{i}({A}_{i}-a{)}^{2}$$

Constraints

$$\sum _{j=1}{S}_{j}=S=1605$$

$$0\le {S}_{j}\le 5$$

## Results

### Current and optimized SA and PCI for MT

Optimization using 2SFCAM and QP improved the demand-weighted SA index (median) from 2.811 to 3.919 and reduced the standard deviation (SD) from 125.625 to 97.290, ranging from 0.000–4,903.194 to 0.000–2,704.246 (Table 1). This indicates that the NES reallocation reduced the SA to MT disparity between each mesh. In the sensitivity analysis, the demand-weighted SA index (median, SD, and range) improved when the travel friction β varied from 0.07 to 0.02. Therefore, reallocation significantly reduces SA disparity even if the distance decay function changes.

**Table 1:** Current and optimized weighted spatial accessibility in meshes

	Current			Optimized		
β	Median	SD	Range	Median	SD	Range
0.07	2.811	125.625	0.000–4,903.194	3.919	97.290	0.000–2,704.246
0.06	3.193	122.928	0.000–4,816.163	4.618	94.725	0.000–2,616.724
0.05	3.615	120.093	0.000–4,698.958	5.348	92.339	0.000–2,555.276
0.04	4.076	116.842	0.000–4,530.576	6.014	90.637	0.001–2,504.584
0.03	4.603	112.839	0.001–4,286.824	6.635	89.404	0.001–2,477.626
0.02	5.237	107.856	0.001–3,948.427	7.139	89.011	0.002–2,479.458

β is the travel friction coefficient. *SD*, standard deviation.

**Fig. 1:**
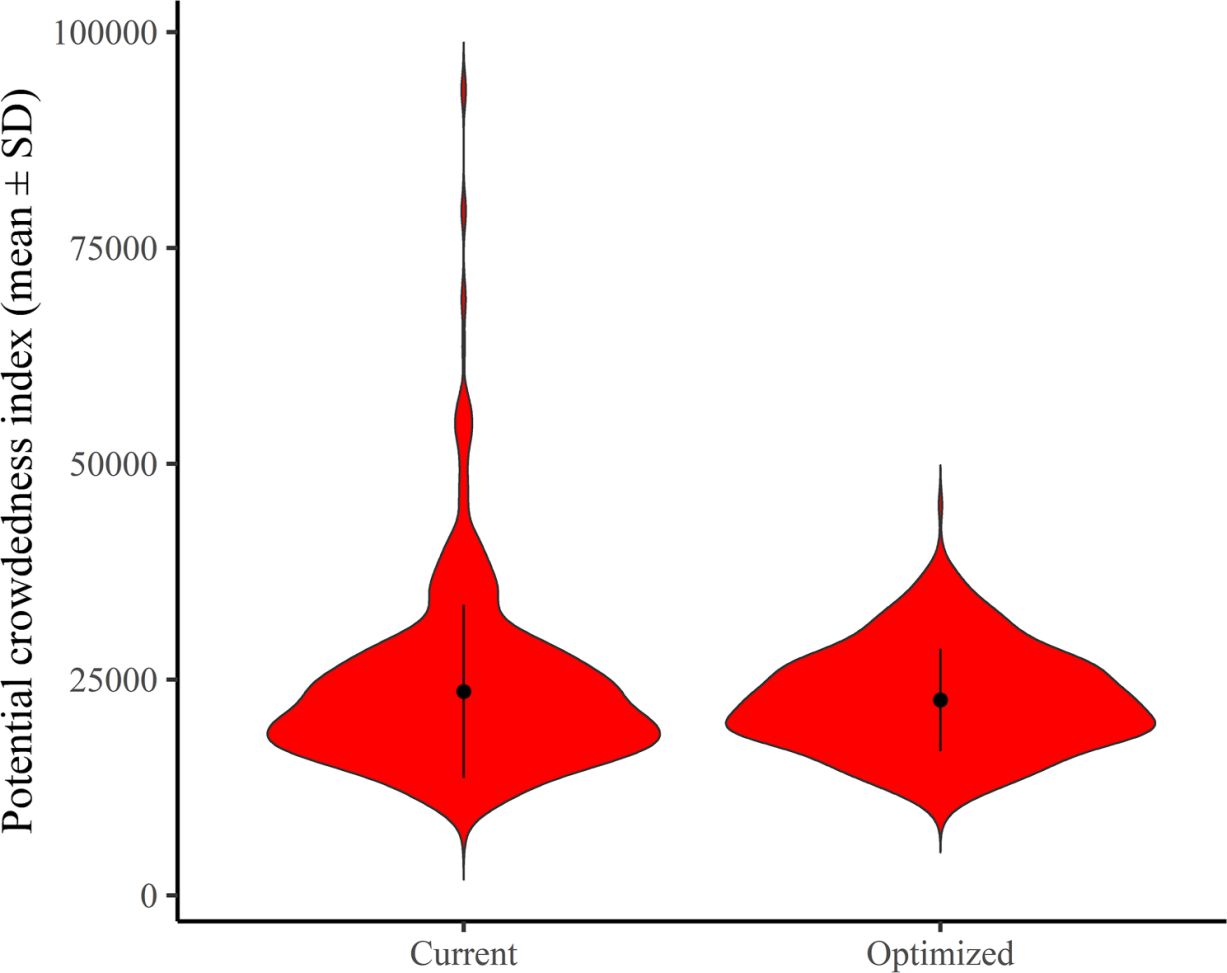
This violin plot shows the distribution of the PCI before and after optimization. The travel friction coefficient, β, was 0.07. The center and straight lines represent the mean and standard deviation, respectively

Next, this optimization reduced the SD and range in PCI from 10,040.36 to 5934.275 and 7045–93,628 to 9624–45,227, respectively. However, the mean PCI score remained unchanged in all the analyses (Table 2). This is an expected result because this setting did not increase the overall number of NES. The number of hospitals with 0.01 or an NES ranged from 366 to 494. As the coefficient of friction β decreases, the number of hospitals with almost no NES increases. Figure 1 shows that this optimization achieved centralization of the PCI distribution.

**Table 2:** Current and optimized PCI in facilities

	Current			Optimized			
β	Mean	SD	Range	Mean	SD	Range	Number of hospitals (Physician ≥ 0.01)
0.07	23,633	10,040.36	7,045–93,628	22,643	5,934.275	9,624–45,227	494
0.06	23,250	9,284.819	7,619–92,840	22,658	6,072.832	9,106–43,412	476
0.05	22,881	8,535.032	6,119–92,617	22,508	5,905.165	7,038–40,342	448
0.04	22,552	7,818.882	4,898–92,318	22,416	5,816.385	6,649–38,389	427
0.03	22,301	7,163.317	4,350–91,937	22,161	5,670.852	6,440–36,890	386
0.02	22,167	6,598.864	4,758–91,500	22,151	5,585.68	6,918–38,465	366

There are currently 662 hospitals in Japan. Here, β is the travel friction coefficient. *SD*, standard deviation.

### Description of PCI at the prefecture-level

**Fig. 2:**
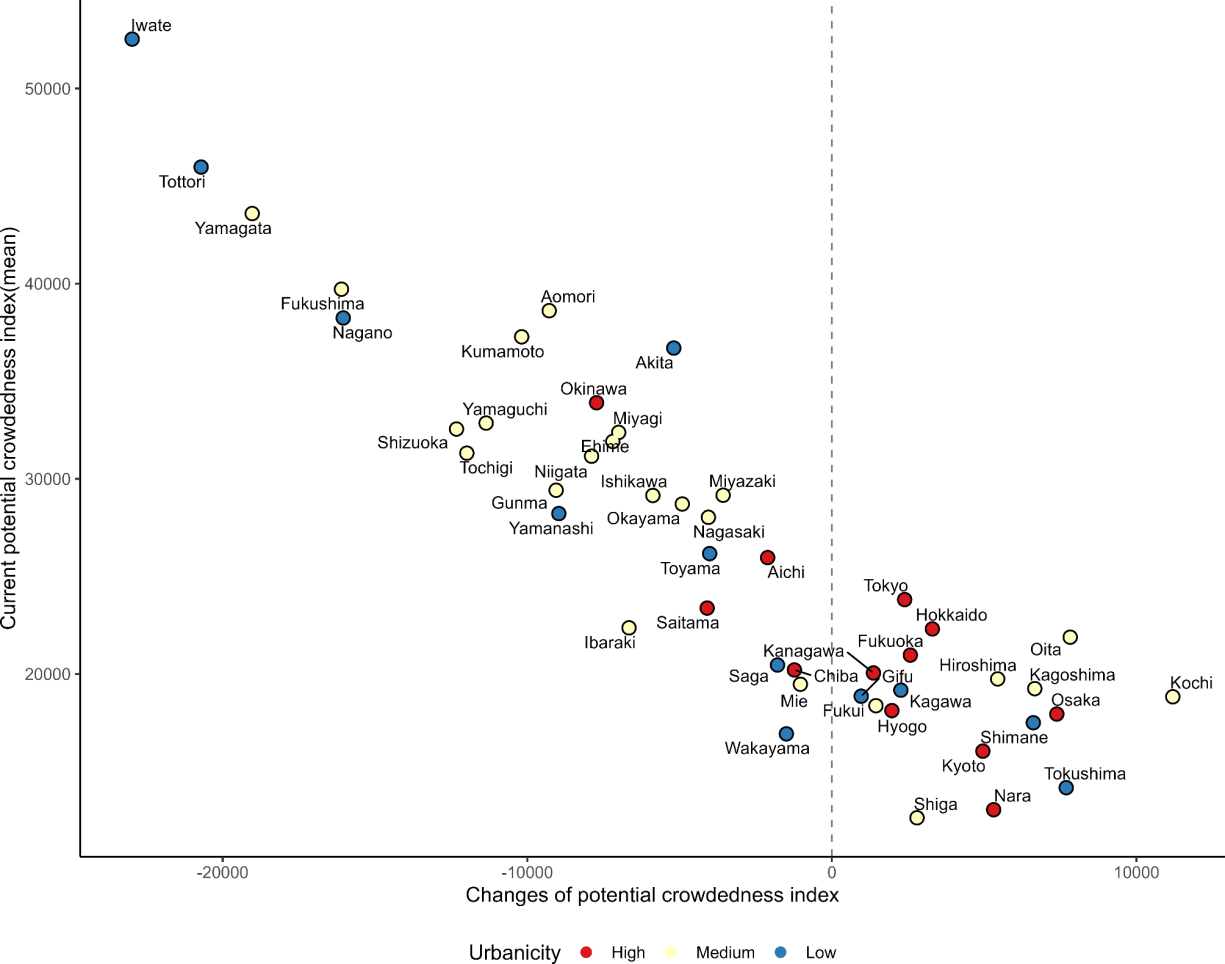
Changes in potential crowdedness index (x-axis) and mean current potential crowdedness index (y-axis) for 47 prefectures. Prefectures were divided into three groups based on the percentage of the population living in Densely Inhabited Districts. The higher the percentage, the more urbanized the area. The top 25% was defined as high, the bottom 25% as low, and the others as medium

**Fig. 3:**
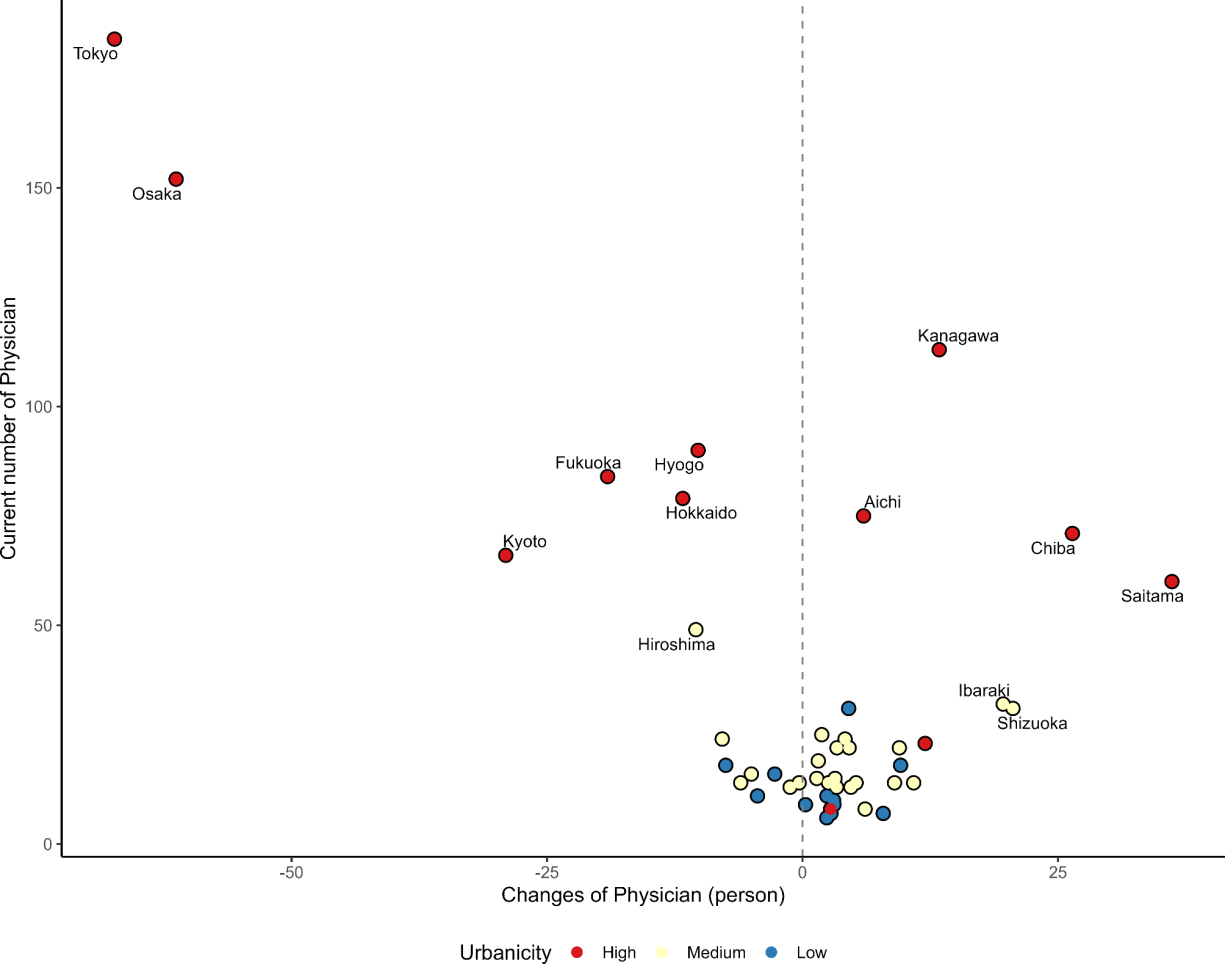
The scatter plot expresses the current number of neuroendovascular specialists and changes in the number of neuroendovascular specialists for 47 prefectures after optimization. The dotted lines represent zero. Prefectures were divided into three groups based on the percentage of the population living in Densely Inhabited Districts. The higher the percentage, the more urbanized the area. The top 25% was defined as high, the bottom 25% as low, and the others as medium

Figure 2 shows the changes in PCI after optimization at the prefecture level. The distribution was downward sloping-to-the-right, and a higher current meant that the PCI tended to decrease post-optimization. Specifically, Iwate, Tottori, and Yamagata, which had high PCI, improved after optimization. Thus, NES reallocation contributed to reducing the disparity in PCI at the prefecture level. The coloring in Fig. 2 represents urbanicity, divided into three segments based on the population proportion of densely inhabited districts. The top 25% were categorized as high urbanicity, the bottom 25% as low urbanicity, and the middle 50% as medium urbanicity. The high urbanicity category initially exhibited good PCI and was clustered around the center. In contrast, the low urbanicity category showed greater variability. Figure 3 shows the changes in NES (person) after optimization and the current number of NES (person). Our optimization simulation indicates that while there is a notable necessity for relocation between urban areas, these changes aim to bridge the urban-rural gap in MT access. This ensures that medical resources are more evenly distributed, benefiting both urban centers and their neighboring prefectures. The top three prefectures with the largest decreases in the number of NES were Tokyo, Osaka, and Kyoto. Conversely, the top three prefectures with the largest increase in the number of NES were Saitama, Chiba, and Shizuoka. Similar to Figs. 2 and 3 is color-coded according to urbanicity. Maps of the prefecture locations and urban population rates are shown in Additional file 1.

## Discussion

This study demonstrated that optimization via the reallocation of NES reduced the disparity between SA and PCI simultaneously. Furthermore, the mean PCI gap has narrowed at the prefectural level. Previous studies with the same optimization method demonstrated the equalization of SA from a demand perspective [16, 17], consistent with our results. Additionally, this result emphasizes the equalization of PCI from a supply perspective. Regional disparities in the stroke medical care system in Japan are an issue in medical policy [27–29], and our results suggest that balancing access to healthcare and the workload of physicians are viable strategies.

The Japanese government developed the “Work Style Reform” initiative to reduce overtime work for physicians [30]. There are concerns that some hospitals will face difficulties in maintaining the medical system because the campaign will cap overtime hours for physicians in 2024. Physicians working overtime is a serious problem in Japan. An investigation in 2019 reported that approximately 40% of physicians worked over 60 h/week, and 10% worked over 80 h/week [31]. In other words, there is a trade-off between “Work Style Reform” and access to healthcare, and optimizing working hours alone will worsen access to healthcare. However, hospitals that operate 24/7 are ideal for an acute stroke care system. To achieve this, several physicians are enrolled in the hospital to maintain the quality of medical care and the working environment of physicians. Under the assumption that no new facilities will be built, we propose bringing physicians from several facilities together. Table 2 shows the number of hospitals after physician staffing conversion. The range was 366–494. This increases the number of physicians per facility because the NES, originally allocated to 662 facilities, is now reallocated to some hospitals. Therefore, reallocating physicians using this model will improve the vulnerability and SA disparities in the healthcare delivery system.

Regarding the factors contributing to MT implementation at the prefectural level, Maeda et al. showed that prefectures with a low density of NES had fewer cases [19]. Prefectures with fewer MT practices (0 to 2.5/100,000 people) were Iwate, Yamagata, Niigata, Tochigi, Saitama, Yamanashi, Shizuoka, Nara, and Ehime [19]. Compared to our results, these prefectures had similarly high PCIs; however, Nara and Saitama differed. This needs to be interpreted with an understanding of the differences in the data sources. First, the previous study used claim data (2015) for the number of MTs and the general population size. Conversely, the PCI we used was calculated using the number of specialists in 2021 as supplied and the estimated population aged ≥ 65 years, as demanded. PCI is simply the ratio of NES to the population aged ≥ 65 years, which is the same as specialist density. Our analysis improves upon previous methods by realistically accounting for the possibility of patients being transported to nearby hospitals. In essence, regions with a high PCI, indicating fewer resources per capita, might still perform fewer MTs, even if they were reported by Maeda et al. to have low MT numbers. This discrepancy could be due to current shortages in NES. Conversely, Saitama and Nara, with low PCI, suggested that the number of MTs may have increased.

This is a strength over classical methods of assessing healthcare resources, such as the physician-to-population ratio at the county level [32]. That is, this index captures the influence of newly allocated physicians and is not limited to the range of the prefecture. Figure 2 demonstrates a consistent improvement in PCI regardless of urbanicity. This improvement is evident even within low-urbanicity areas, exhibiting a mix of good and poor PCI areas. This pattern extends to the prefectural level, where adjacent low-urbanicity prefectures interact with high-urbanicity prefectures, resulting in shared medical resources. This indicator effectively captures this situation. For instance, strategically assigning physicians in Saitama and Chiba, which are high-urbanicity areas, can positively impact the PCI of neighboring prefectures, such as Ibaraki, Tochigi, and Gunma. Our findings also suggest that the gap between SA and PCI will be narrowed by reallocating NES from Tokyo and Osaka to Iwate, Yamagata, Saitama, and Kanagawa. The caveat in implementing this relocation model is that some facilities and areas will experience increased workloads and less accessibility unless the total supply increases. While our model aims to optimize access on a larger scale, we acknowledge that certain regions might experience shifts that require further considerations. Specifically, areas with currently good access and a low PCI facility may experience adjustments. Our goal is to ensure that no region is left vulnerable due to these shifts, and we advocate for regular reviews post-reallocation to ensure equity and quality in stroke care.

In the sensitivity analysis, the uncertainty in the results of this study was evaluated by varying the friction coefficient in the distance decay function and emergency transport time. Decreasing the friction coefficient β to 0.07–0.02 decreases the worth of EMS time in stroke outcomes, meaning that the criteria for choosing a destination can be factors other than time (e.g., resources and congestion). Selecting a hospital from a wider range of locations where the stroke occurred will reduce the disparity between SA and PCI. Thus, changing the emergency transport system reduces physicians’ unbalanced workloads. The time to reperfusion is critical to the clinical outcome of stroke patients [33–35], and a critical time limit for clinically significant treatment effects needs to be defined.

### Strength and limitations

Our findings provide evidence that physician reallocation using the 2SFCAM and QP can improve the disparities between SA and PCI. It can be applied in other countries and medical departments, considering the differences in emergency transport methods and insurance systems. However, this study had several limitations. First, demand points are based on the population aged ≥ 65 years and residential location. Thus, it may underestimate demand in an urban area where the large population aged < 65 years and the difference between day and night populations are large. For example, the daytime population of Tokyo was approximately 20% higher than the permanent population in 2020 [36]. Second, this study did not consider regional differences in stroke incidence or risk factors for stroke (e.g., hypertension, smoking rates, and diabetes). Therefore, the application of demand weighted by regional differences in stroke incidence will develop this model. Third, we defined NES as the supply, and the number of NES assigned to each facility was fixed at 0–5. To perform MT, other medical resources are required, such as medical equipment and interventional radiology units [37]. The range of necessary resources for MT varies across facilities, and the mere presence of NES is not the sole determinant. A simulation incorporating the supply capacity of each facility is required for implementation. Assuming that the number of people to be allocated according to the medical equipment of each facility provides a more appropriate reallocation. Therefore, the first step in the reallocation model is properly selecting candidate facilities. In our study, the facility’s full readiness for MT is validated by the PSC certification criteria, providing a holistic benchmark. As the field advances, we will strive to update our selection of candidate facilities when more refined criteria for MT emerge in the future. Fourth, the distance decay function and reachable area were fixed for all facilities in this study. To capture a suitable distance decay function, these settings should be flexible, depending on the facility or area. However, the setting for each facility is not always rational because the emergency medical system relies on daily cooperation among multiple hospitals. Finally, building a new facility in a new place would yield quite different results. Optimization methods that include facility location have been proposed as an expansive method to achieve equity in healthcare [38].

## Conclusions

Reallocation of NES by applying the QP and 2SFCAM reduced regional disparities in SA and PCI in stroke treatments, as well as disparities between facilities. Our findings contribute to planning health policies to realize an equitable healthcare system.

### Electronic supplementary material

Below is the link to the electronic supplementary material.

Supplementary Material 1

## Data Availability

The datasets used and/or analyzed during the current study are available from the corresponding author upon reasonable request.

## List of abbreviations

SA: spatial accessibility
2SFCAM: two-step floating catchment area method
PCI: potential crowdedness index
QP: quadratic programming
MT: mechanical thrombectomy
NES: neuroendovascular specialist
SD: standard deviation
