# Supplementary material for: Optimal allocation of physicians improves accessibility and workload disparities in stroke care
Source: Int J Equity Health. 2023 Nov 7;22:233. doi: 10.1186/s12939-023-02036-9 (PMC10631210; doi:10.1186/s12939-023-02036-9)
Supplement: Supplementary file 1 — Supplementary Material 1 [file 12939_2023_2036_MOESM1_ESM.docx]

Supplementary

Certification criteria of primary stroke center (PSC)^1)^

1. Stroke physicians are available 24 hours / 7 days a year to response stroke patients from the request of local medical institutions and emergency medical services and can treat (including intravenous rt-PA therapy) as soon as possible.
2. Head computed tomography or magnetic resonance imaging examinations, general blood tests and coagulation tests, and electrocardiogram tests can be performed.
3. Has a stroke unit (SU).
4. Physicians engaged in stroke treatment (excluding first-year trainees, who do not need to be full-time) work 24 hours/7 days a year.
5. Has at least one full-time stroke specialist.
6. A system in which a neurosurgeon can promptly respond when neurosurgical procedures are required.
7. It is desirable that mechanical thrombectomy can be performed, if not possible, emergency transfer of patients who are eligible for mechanical thrombectomy will be conducted between nearby primary stroke centers where mechanical thrombectomy therapy is always available. have a procedure manual.
8. Management the quality of stroke medical care by obtaining clinical indicators.
9. The Japan Stroke Society. About of Primary Stroke Center (PSC) certification.　 <https://www.jsts.gr.jp/facility/psc/index.html> [Accessed 2023 Sep-13]

Figure S1

**
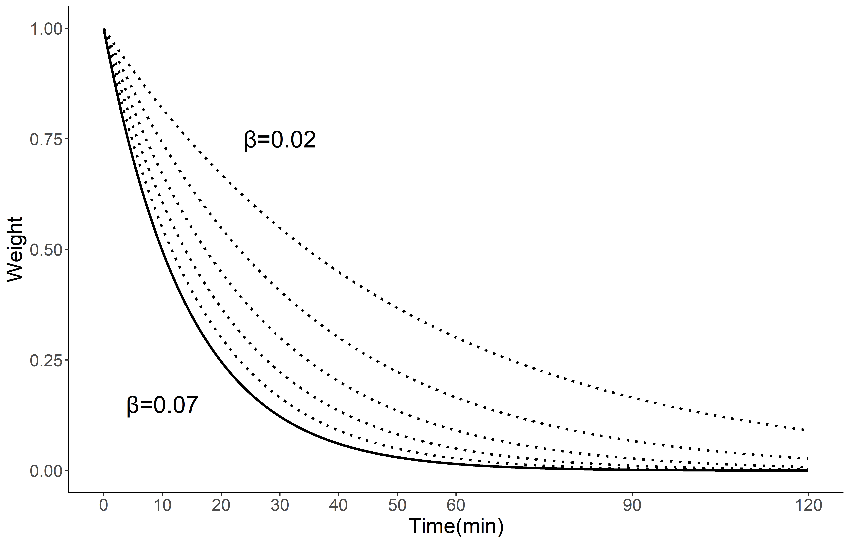
**

Figure legend:　Fig S1 expressed the distance decay function. β was a travel friction coefficient. The travel time between mesh and facility is converted to a value between 0-1 based on the distance decay function.

Figure S2


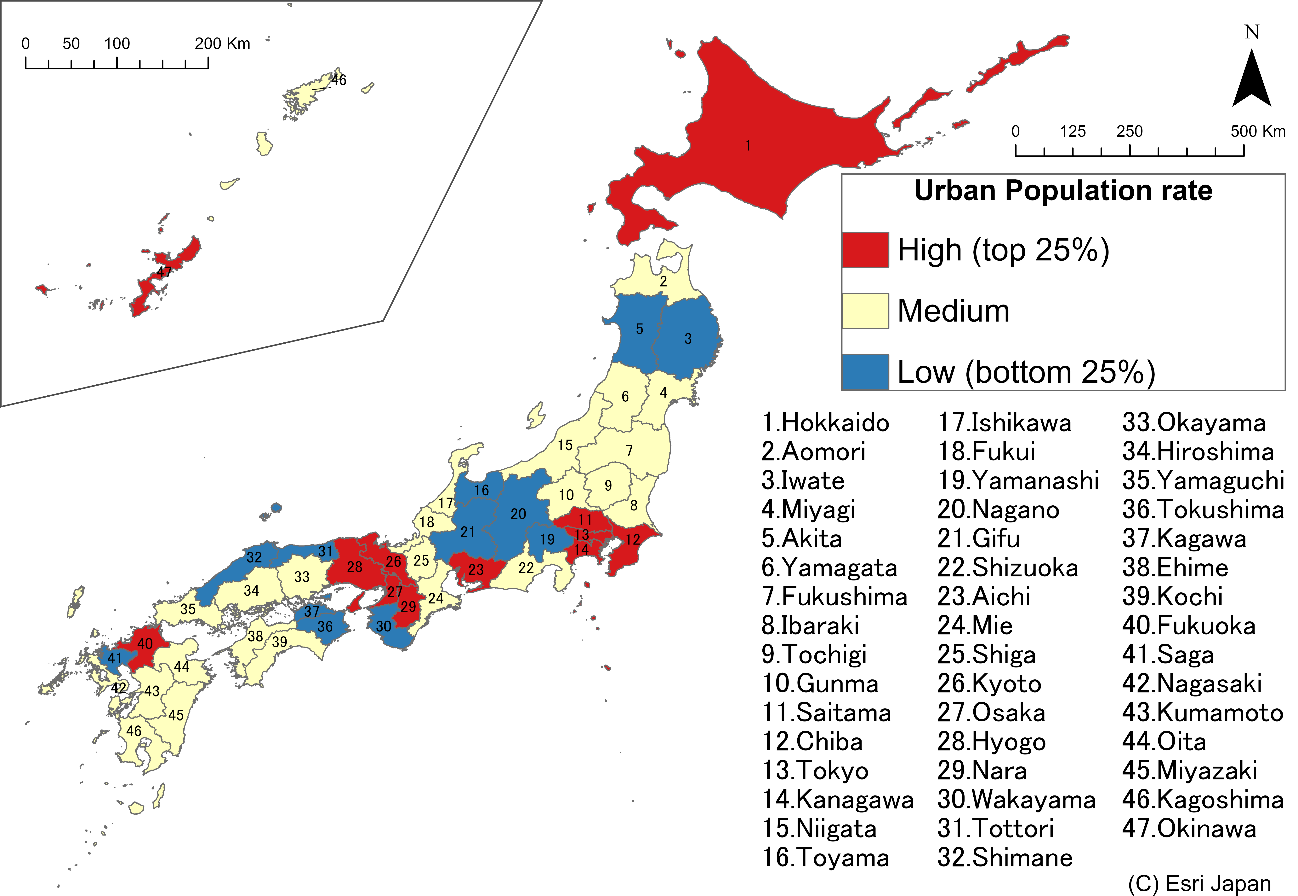


This map was created using numerical maps (basic land information) published by the Geospatial Information Authority of Japan and ESRI Japan's national municipal boundary data.

The urban population rate is the ratio of the population residing in the densely inhabited district ^2)^ to the total population of the prefecture. Higher proportions indicate more urbanization.

1. What is a Densely Inhabited District, Statistic Bureau of Japan. <https://www.stat.go.jp/english/data/chiri/did/1-1.html> [Accessed 2023 Aug-10]
